# Systematic literature review on the neonatal outcome of preeclampsia

**DOI:** 10.11604/pamj.2022.41.82.31413

**Published:** 2022-01-31

**Authors:** Temitope Folasade Atamamen, Nyi Nyi Naing, Jaiyeola Adedayo Oyetunji, Nadiah Wan-Arfah

**Affiliations:** 1 Faculty of Medicine, Universiti Sultan Zainal Abidin, Medical Campus, Jalan Sultan Mahmud, Kuala Terengganu, Teregganu, Malaysia,; 2 Department of Health Information Management, Federal Medical Centre, Birnin Kebbi, Kebbi State, Nigeria,; 3 Johns Hopkins Program for International Education in Gynecology and Obstetrics (Jhpiego), Abuja, Nigeria,; 4 Department of Obstetrics and Gynaecology, Federal Medical Centre, Birnin Kebbi, Kebbi State, Nigeria,; 5 Faculty of Health Sciences, University Sultan Zainal Abidin, Kuala Nerus, Terengganu, Malaysia

**Keywords:** Neonatal, outcomes, preeclampsia

## Abstract

Preeclampsia is a pregnancy-specific multisystem disorder that is a leading cause of maternal and foetal/neonatal morbidity and mortality. Thus this systematic review aims to identify the neonatal outcomes of preeclamptic patients. A systematic literature review of works published between January 2015 and March 2021 written in the English language and freely accessed online were used considering the PRISMA guidelines. The results from the search were managed using the endnote X7 software and extracted data from the full articles were documented in Microsoft Word. The neonatal outcomes of preeclampsia identified are; preterm birth, stillbirth, low birth weight (LBW), low Apgar score, intrauterine growth reduction (IUGR), neonatal intensive care unit (NICU) admission are foetal/neonatal outcomes of preeclampsia and were subsequently classified into six groups according to the similarities of their outcome; group 1: death related neonatal outcomes, group 2: weight-related neonatal outcomes, group 3: prematurity related neonatal outcomes, group 4: respiratory related neonatal outcomes, group 5: injury-related neonatal outcomes, and Group 6: internal organ related outcome. The magnitude of occurrence of the classified neonatal outcomes is; respiratory-related neonatal outcome, death-related neonatal outcome, weight-related neonatal outcome, prematurity related neonatal outcome, internal related neonatal outcome and injury-related outcome in that sequence. All round interventions to improve neonatal morbidity and mortality of preeclamptic mothers should be targeted in addition to adequate provision of health/ medical resources for the tending of preterm neonates.

## Introduction

Preeclampsia (PE) is a pregnancy-specific multisystem disease that is the main cause of maternal and foetal/neonatal morbidity and mortality globally [1]. Preeclampsia is a pregnancy-specific hypertensive syndrome related to considerable morbidity and mortality in mothers and neonates, even though underreporting has brought about underestimated incidence in some places, PE is a disorder that health professionals need to understand how to clinically control to save the life of the mother and child [2,3]. Preeclampsia (PE) is a hypertensive disorder characterized via a widespread endothelium in the mother and consequences in high blood pressure of 140/90mmHg or more, with proteinuria occurring after 20 weeks of gestation. According to [4] the International Society for the Study of Hypertension in Pregnancy (ISSHP) in 2014 defines pre-eclampsia as de-novo hypertension present after 20 weeks of gestation combined with proteinuria (>300mg/day) and other maternal organ dysfunctions such as renal insufficiency, liver involvement, neurological or hematological complications, uteroplacental dysfunction, or foetal growth restriction. Severe preeclampsia (severe PE) in accordance to [2] was diagnosed where the blood pressure used to be 160/110mmHg and above besides thrombocytopenia, progressive renal dysfunction, impaired liver function, pulmonary oedema, and new-onset cerebral or visible disturbances with the presence of extreme and chronic right top quadrant or epigastric pain no longer responsive to medication and other causes excluded.

Though [5] stated that the international society for the study of hypertension in pregnancy (ISSHP) has previously published an assertion documenting severe preeclampsia, however, the authors agree with the position of the American College of Obstetricians and Gynecologists (ACOG) that preeclampsia may come to be a predominant risk to mother and child at any stage, and classification into mild or extreme disease can be inaccurate or deceptive to less experienced clinicians. American college of obstetricians and gynecologists has eliminated the diagnosis of severe preeclampsia and instead discusses preeclampsia with or without severe features, a sensible clinical approach. Preeclampsia ranks 2^nd^ or third in the global ranking of causes of maternal morbidity and mortality. Preeclampsia has an incidence of 2-10% of all pregnancies, with a higher incidence rate in developing countries and low settings. It is stated that 10% of women have high blood pressure (HBP) during pregnancy and 2-5% result in PE [4]. Newborns of women with PE have approximately twice the danger of neonatal death [6] buttressing the devastating outcomes of PE on foetuses and newborns as noted by [4,7] certain regions of the world, particularly Africa, are still struggling to meet the millennium development goal four (MDG4), which intends to minimize infant mortality ratio globally. Therefore, this review is necessitated to fill this gap in the literature, as there is still a dearth of information on the rate and causes of neonatal mortality and morbidity. Hence, this review aims to identify the neonatal outcomes of PE, which is a known cause of neonatal mortality and morbidity. It is against this background that this study is being undertaken to uncover the neonatal outcomes of preeclampsia.

## Methods

The systematic literature review was done on articles of neonatal outcomes of preeclampsia globally, freely available articles on the internet were accessed. The results of these studies were reported following preferred reporting items for systematic review and meta-analysis statement (PRISMA) 2020 guidelines.

**Inclusion and exclusion:** Temitope Folasade screened titles, and abstracts of identified citations for potential inclusion in the review, and full texts were sought for relevant articles. Inclusion criteria for the search were published articles and electronic articles from January 2015 to March 2021 related to the neonatal outcome of preeclampsia research globally, which were freely accessible. These included articles from primary studies, and review articles (systematic review or narrative review). Studies before 2015 and non-English language articles were excluded.

**Figure 1:**
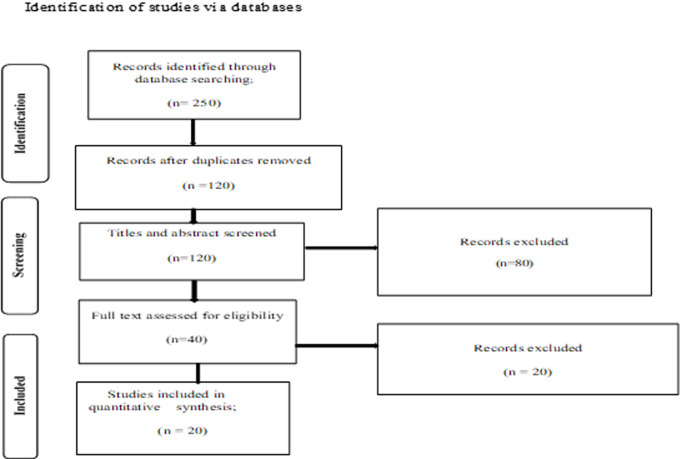
details of study flow in different stages of the review

**Study selection:** selection of articles was performed in two stages. In the first stage, the titles and abstracts of all resources based on the inclusion criteria and search terms were screened. Selected titles and abstracts were then screened and checked whether the content potentially answered the review questions. Irrelevant abstracts were excluded and the researcher then retrieved full articles of the selected abstracts. In the second stage, full articles were screened to identify items related to the objectives of the review. Similar to the first stage, full articles were reviewed to confirm if they met the objectives of the review. The selection of articles was done using the PRISMA flow diagram as shown in Figure 1.

**Search strategy and information sources:** a comprehensive search to identify primary studies, reviews, grey literature on neonatal outcomes of Preeclampsia was initially conducted to capture studies published in the last five years. However, this period was extended till March 2021 to update the literature search before the final analysis and writing were done. The search was performed using different electronic databases (PubMed, and Google Scholar). The search strategy was developed based on search terms. Boolean operators (or, and,) were used to combine the keywords (neonatal, outcomes, preeclampsia) and related terms during the literature search.

**Data extraction:** articles were excluded if they were not relevant and did not describe neonatal outcomes of Preeclampsia and the objectives of the review and if the date of publication was outside January 2015 - March 2021. Relevant articles were then assessed to answer the review questions. Study characteristics extracted from publications include the following; author name, study design, year of publication, country of publication, target population (age), neonatal outcomes of preeclampsia and limitations or gaps of the study. The results from the search were managed using the end note X7 software, and extracted data from the full articles were documented in Microsoft Word.

**Quality appraisal:** quality appraisal was performed on all freely accessed qualitative and quantitative studies published during the search period. Included studies were appraised for relevance. No studies were removed as a result of the quality appraisal.

**Data analysis:** statistical analysis was performed by a biostatistician using the Excel 2013 version. Descriptive analysis was performed for variables relating to the year of publication and number of studies, percentages of continents represented in the study, percentage of each neonatal outcome of preeclampsia and the six groups into which they were classified. The percentage of each study design was also calculated.

## Results

A total of 250 titles and abstracts were found and after careful screening and removal of the duplicates, 20 studies were shortlisted for this review. These 20 documents were included in the charting process and the characteristics of the studies are shown in Table 1. In terms of the number of publications from 2015-2021, the review shows 2015(n=1), 2016 (n=2), 2017(n=3), 2018(n=4), 2019 (n=3), 2020 (n=6) and 2021(n=1). While significant works have been carried out on PE, however, few studies have been done on the neonatal outcomes of PE of which there was no significant difference in the neonatal outcomes of PE from reviewed literature across the continents Table 2.

**Table 1:** eligibility criteria employed

Inclusion criteria	Exclusion criteria
Study identified as quantitative or qualitative studies on neonatal outcomes of preeclampsia	Studies focusing on other neonatal issues and specialities different from neonatal outcomes of preeclampsia
Freely accessed articles	Non-human subjects
English language	Non-neonate studies
Published from 1^st^ January 2015 to March 2021 (online or in print)	Non-human subjects

**Table 2:** year of publication and number of studies

Year of publication	Number of studies n(%)
2015	1 (5)
2016	2 (10)
2017	3 (15)
2018	4 (20)
2019	3 (15)
2020	6 (30)
2021	1 (5)
**Total**	20 (100)

**Study characteristics:** as earlier stated, the 20 articles reviewed were published between the years 2015 and 2021. Twenty three (23) neonatal outcomes of preeclampsia were identified from studies carried out in four continents and 19 countries. Most of the studies are from Africa (52.63%), four publications from Ethiopia [1-4], two publications from Zimbabwe and Ghana respectively [5-7], Kenya [8], and Egypt [9] one publication each. Five publications from America (26.31%), USA [10], Canada 8 [11], West-Indies [12], Haiti [13], and Washington D.C [14]. Two publications from Asia (10.53%), India [15] and China [16] and also Europe (10.53%), France [17] and Norway [18] respectively. All articles reviewed are published in the English language. Most of the articles reviewed 12(60%) were retrospective study, cross-sectional study 3(15%), prospective study 3(15%), case-series 1(5%) and write-up 1(5%).

**Neonatal outcome:** from the 20 articles reviewed on the neonatal outcome of preeclampsia, 23 neonatal outcomes were identified. The outcomes were presented in Table 3 and observed in the following order: low birth weight (LBW)/ small for gestational age (SGA) was the most observed with 11 authors attesting to it, followed by prematurity [3,4,8,11-13,15,17,19] with nine authors confirming it. Following this are outcomes attested to by eight authors such as stillbirth [1-4,6,9,13,19], low APGAR score [1,2,4-6,11,12,14] and early mortality/neonatal death [2,3,6,8,12, 14,15,20]. Seven authors identified neonatal intensive care unit (NICU) admission [1,3,6,8,12,14,19] and foetal growth restriction (FGR)/intrauterine growth restriction (IUGR) [1,3-5,10,15,20] as outcomes of neonatal preeclampsia. Respiratory disease syndrome (RDS) was observed by four authors as an outcome [3,8,15,19] followed by birth asphyxia as established by three authors. This is also followed by outcomes such as abortion [2,6] and placental/birth injuries [14,16] as noted by two authors respectively. Neonatal outcomes identified by single authors are; intracranial haemorrhage [10], jaundice [8], placental abruption [11], need for resuscitation, small bronchopulmonary dysplasia [17], congenital heart defects (CHD)[18], grade III/IV intraventricular haemorrhage (IVH), transient tachypnoea of the newborn, pneumonia, necrotizing enterocolitis (NEC), ventilator use, and neonatal sepsis [14] Table 4.

**Table 3:** neonatal outcomes of preeclampsia and authors

S/N	Author and year	Paper title	Study design and country	Neonatal outcomes of preeclampsia
1	Kartik K Venkatesh *et al* (2020)	Adverse maternal and neonatal outcomes among women with preeclampsia with severe features <34 weeks gestation with versus without comorbidity	Retrospective study (USA)	foetal growth restriction (FGR), Intracranial or periventricular haemorrhage
2	Charity NdwigaI *et al* (2020)	Clinical presentation and outcomes of preeclampsia and eclampsia at a national hospital, Kenya: a retrospective cohort study	Retrospective study (Kenya)	Mortality, prematurity, low birth weight (LBW), NICU admission, respiratory distress syndrome (RDS), jaundice
3	Min Xue Shen *et al* (2017)	Comparison of risk factors and outcomes of gestational hypertension and pre-eclampsia	A prospective study (Canada)	Preterm, low Apgar score, placental abruption
4	Mekoya D Mengistu (2020)	Feto-maternal outcomes of hypertensive disorders of pregnancy in Yekatit-12 Teaching Hospital, Addis Ababa: a retrospective study	Retrospective study (Ethiopia)	Low APGAR score, stillbirth, low birth weight (LBW), FGR, NICU admission, low APGAR score, need for resuscitation
5	Solwayo Ngwenya (2017)	Severe preeclampsia and eclampsia: incidence, complications, and perinatal outcomes at a low-resource setting, Mpilo Central Hospital, Bulawayo,	Retrospective study (Zimbabwe)	Stillbirth, preterm, LBW, NICU admission, RDS,
6	Eshetu Seyom (2015)	Maternal and foetal outcome of pregnancy related hypertension in Mettu Karl Referral Hospital	Retrospective study (Ethiopia)	Stillbirth, Neonatal death Abortion, LBW, low APGAR score
7	Lemi Belay Tolu *et al* (2020)	Maternal and perinatal outcome of preeclampsia without severe feature among pregnant women managed at a tertiary referral hospital in urban Ethiopia	A prospective study (Ethiopia)	Stillbirth, Early neonatal death, preterm, LBW, IUGR, NICU admission, RDS
8	Edward T Dassah *et al* (2020)	Maternal and perinatal outcomes among women with hypertensive disorders in pregnancy in Kumasi, Ghana	Cross-sectional study (Ghana)	Stillbirth, Abortion/perinatal mortality, preterm, LBW, NICU admission, low APGAR score
9	Subrat Panda *et al* (2021)	Maternal and perinatal outcomes in hypertensive disorders of pregnancy and factors influencing it: a prospective Hospital-based study in Northeast India	A prospective study (Northeast India)	Neonatal death, IUFD, preterm, FGR, RDS
10	Kerri-Ann McKenzie *et al* (2018)	A retrospective study of neonatal outcome in preeclampsia at the University hospital of the West Indies: a resource-limited setting	Retrospective study (west Indies)	Neonatal death, preterm, LBW/SGA, NICU admission, low APGAR score
11	Misganaw Fikirie Melese *et al* (2019)	Perinatal outcomes of severe preeclampsia/eclampsia and associated factors among mothers admitted in Amhara region referral hospitals, North West Ethiopia	Cross-sectional study (Ethiopia)	Stillbirth, abortus, preterm, LBW, IUGR, low APGAR score, birth asphyxia
12	Pauline Dravet-Gounot *et al*(2018)	Bronchopulmonary dysplasia in neonates born to mothers with preeclampsia: impact of small for gestational age	Retrospective study (France)	Preterm neonates, SGA, small bronchopulmonary dysplasia in very preterm neonates born
13	Yanyan Ni *et al* (2016)	Comparison of indications of pregnancy termination and prognosis of mothers and neonates in early- and late-onset preeclampsia	Retrospective study (China)	Severe placental and perinatal injuries
14	Takura Innocent Kanonge *et al* (2018)	Hepatic rupture from haematomas in patients with pre-eclampsia/eclampsia: a case series	Case series (Zimbabwe)	Macerated stillborn infant
15	Matthew Bridwell *et al* (2019)	Hypertensive disorders in pregnancy and maternal and neonatal outcomes in Haiti	Retrospective study (Haiti)	Stillbirth, preterm, SGA,
16	Kwame Adu-Bonsaffoh *et al* (2017)	Perinatal outcomes of hypertensive disorders in pregnancy at a tertiary hospital in Ghana	Cross-sectional study (Ghana)	LBW, IUGR, low apgar score, birth asphyxia
17	Kristoffer Brodwall *et al*(2016)	Possible Common aetiology behind maternal preeclampsia and congenital heart defects in the child: a cardiovascular diseases in Norway project study	Retrospective study (Norway)	Congenital Heart Defects (CHD)
18	Antonette T Dulay *et al*(2018)	Preeclampsia and eclampsia	Write-up	foetal death, FGR
19	Ahmad Mahran *et al* (2017)	Risk factors and outcome of patients with eclampsia at a tertiary hospital in Egypt	Retrospective study (Egypt)	Stillbirth; ante-natal and intrapartum deaths and early neonatal death
20	Elizabeth M Coviello *et al* (2019)	Early preterm preeclampsia outcomes by intended mode of delivery	Retrospective study (Washington, DC)	Neonatal death, NICU admission, low APGAR score, RDS, asphyxia, grade III/IV intraventricular haemorrhage (IVH); transient tachypnoea of the newborn, pneumonia, birth injury, necrotizing entero colitis (NEC), ventilator use, neonatal sepsis

**Table 4:** neonatal outcomes of preeclampsia, number of observations, and percentages

S/N	Neonatal outcomes of preeclampsia	Number of observations	Percentage (%)
1	Low birth weight (LBW)/small for gestational age (SGA)	11	13.58
2	Prematurity/preterm births	9	11.11
3	Stillbirth	8	9.88
4	Low apgar score	8	9.88
5	Early mortality/neonatal death	8	9.88
6	Neonatal intensive care unit (NICU) admission	7	8.64
7	Foetal growth restriction (FGR)/intrauterine growth restriction (IUGR)	7	8.64
8	Respiratory disease syndrome (RDS)	4	4.94
9	Birth asphyxia	3	3.70
10	Abortion	2	2.47
11	Placental/birth injuries	2	2.47
12	Intracranial or periventricular haemorrhage	1	1.23
13	Jaundice	1	1.23
14	Placental abruption	1	1.23
15	Need for resuscitation	1	1.23
16	Small bronchopulmonary dysplasia	1	1.23
17	congenital heart defects (CHD	1	1.23
18	Grade III/IV intraventricular haemorrhage (IVH)	1	1.23
19	Transient tachypnoea of the newborn,	1	1.23
20	Pneumonia	1	1.23
21	Necrotizing enterocolitis (NEC)	1	1.23
22	Ventilator use	1	1.23
23	Neonatal sepsis	1	1.23

**Broad categories of neonatal outcomes:** the above 23 neonatal outcomes were further categorised into six (6) major groups in this study based on their similarities of the outcomes and the groups are classified as follows: group 1: death related neonatal outcomes which comprises MSB/FSB, abortion and early mortality/ neonatal death; group 2: weight-related neonatal outcomes which includes LBW/SGA and IUGR/FGR; group 3: prematurity related neonatal outcomes which consists of neonatal intensive care unit (NICU) admission and preterm birth; group 4: respiratory related neonatal outcomes are; low APGAR score, respiratory disease syndrome (RDS), ventilator use, small bronchopulmonary dysplasia, pneumonia and transient tachypnoea; group 5: injury-related neonatal outcomes includes intracranial haemorrhage, intraventricular haemorrhage and placental/ perinatal injuries; group 6: internal organ related outcome which is the last group comprises jaundice, necrotizing enterocolitis, placental abruption, need for resuscitation, congenital heart disease (CHD). From the grouping above and review of literature, the percentage of each group of neonatal outcomes was determined by the use of the Microsoft Excel 2013 version (Table 5). Group 4 which is respiratory-related neonatal outcome has the highest percentage 23.46%, followed by group 1, death-related neonatal outcome, and group 2 weight-related neonatal outcome with a percentage of 22.22% respectively. Next is prematurity-related outcome classified in group 3 with the percentage of 19.755 and this was followed by group 6 the internal organ-related neonatal outcome 7.41%. The category with the least percentage in group 5, the injury-related neonatal outcome, which is 4.94%.

**Table 5:** groups of neonatal outcomes and percentages

Group	Neonatal outcomes	Number of outcomes	Percentages	Group total percentage (%)
1	Abortion	2	2.47	
	Stillbirth	8	9.88	
	Early mortality/neonatal death	8	9.88	22.22
				
2	Low birth weight (LBW)	11	13.58	
	Intrauterine growth restriction (IUGR)/foetal growth restriction (FGR)	7	8.64	22.22
				
3	Neonatal intensive care unit (NICU) admission	7	8.64	
	Preterm birth	9	11.11	19.75
				
4	Low APGAR score	8	9.88	
	Birth asphyxia	3	3.70	
	Respiratory disease syndrome (RDS)	4	4.94	
	Ventilator use	1	1.23	
	Small Bronchopulmonary dysplasia	1	1.23	
	Pneumonia	1	1.23	
	Transient tachypnoea	1	1.23	23.46
				
5	Intracranial haemorrhage	1	1.23	
	Grade III/IV Intraventricular haemorrhage	1	1.23	
	Placental/perinatal injuries	2	2.47	4.94
				
6	Jaundice	1	1.23	
	Necrotizing enterocolitis	1	1.23	
	Placental abruption	1	1.23	
	Need for resuscitation	1	1.23	
	Congenital heart disease (CHD)	1	1.23	
	Neonatal sepsis	1	1.23	7.41

## Discussion

This systematic review suggests that preeclampsia (PE) and its neonatal outcomes occur around the globe, and it is still a concern to all, especially African countries which have the highest frequency of articles. The most-reported neonatal outcome of PE is LBW/SGA and the least commonly reported are intracranial hemorrhage, jaundice, placental abruption, need for resuscitation, CHD, neonatal sepsis, small bronchopulmonary dysplasia in very preterm neonates born, and ventilation use. Although quite a number of studies have been published on neonatal outcomes of PE, it seems much attention is given to some salient outcomes and the exceptional outcomes [14] are not documented or recorded by clinicians. Although the primary goal of obstetricians is to deliver infants who are functionally mature and adaptable to the extrauterine domain without the need for intensive care, a growing body of evidence indicates that neonates born to preeclamptic patients are physiologically immature compared to infants born at term and are at significant risk for a wide range of complications because of associated neonatal morbidity and mortality [21,22]. Some researchers acknowledge the difference between severe preeclampsia and non-severe preeclampsia [1], early onset of preeclampsia, and late-onset of preeclampsia [8,14,19] but the difference in their neonatal outcomes are not distinguished for awareness and education of caregivers who attend to preeclamptic patients to know how to monitor both the patients and the foetus in the womb to deliver physiologically matured infants thereby reducing the rate of neonatal morbidity and mortality.

Research is an imperative aspect of the health care system which seeks answers to problems, as well as increases the knowledge on how health care providers would attend to these patients. It is observed that studies on risk factors of PE are more readily available than outcomes of PE, research attention needs to be drawn to understanding more of clinical, biochemical, and radiological neonatal outcomes of PE. Such research could offer useful insights into understanding this issue(s). The number of the identified neonatal outcome of PE indicates that PE is a medical condition that should be handled with special care and appropriate knowledge to avert or reduce the high rate of neonatal morbidity and mortality. The awareness of these neonatal outcomes by both pregnant women, appropriate health workers, policymakers, and the community at large will go a long way in preserving the lives of neonates whose mothers are affected by PE. A relentless effort is hereby advocated to be put in place on the community and national scale to improve awareness of the neonatal outcomes of PE and also in checking on the health of the foetus whenever preeclamptic patients come in for antenatal appointments or any other clinical appointment in the hospital to ensure their wellbeing.

## Conclusion

Preeclampsia is a major complication in pregnancy with significant consequences for both mother and foetus. Comprehensive interventions to improve neonatal morbidity and mortality of preeclamptic mothers should be pursued in addition to adequate provision of resources for the tending of preterm neonates, most especially in developing countries with high rates of preeclampsia. It has been observed that most studies focused more on the outcomes /prognostic factors of the mother, and not many studies have been carried out on the outcomes of PE and E on the child. More PE and E outcomes research needs to be conducted, particularly on affected children in developing countries and areas predisposed to having preeclamptic patients.

### What is known about this topic

- The outcome of preeclampsia is both maternal and neonatal;
- Preeclampsia is a major cause of maternal morbidity and mortality.

### What this study adds

- Preeclampsia is also a major cause of neonatal morbidity and mortality;
- Preeclampsia is a major cause of perinatal and neonatal morbidity;
- Preeclampsia can be a major threat to the foetus at any stage.
